# Biomedical graduate student experiences during the COVID-19 university closure

**DOI:** 10.1371/journal.pone.0256687

**Published:** 2021-09-16

**Authors:** Janani Varadarajan, Abigail M. Brown, Roger Chalkley

**Affiliations:** The Office of Biomedical Research Education and Training, Vanderbilt University School of Medicine, Nashville, Tennessee, United States of America; Universidade de Brasilia, BRAZIL

## Abstract

COVID-19-associated university closures moved classes online and interrupted ongoing research in universities throughout the US. In Vanderbilt University, first year biomedical sciences PhD students were in the middle of their spring semester coursework and in the process of identifying a thesis research lab, while senior students who had already completed the first year were at various stages of their graduate training and were working on their thesis research projects. To learn how the university closure and resulting interruptions impacted our students’ learning and well-being, we administered two surveys, one to the first year students and the other to the senior students. Our main findings show that the university closure negatively impacted the overall psychological health of about one-third of the survey respondents, time management was the aspect of remote learning that caused the highest stress for close to 50% of the students, and interaction with their peers and in-person discussions were the aspects of on-campus learning that students missed the most during the remote learning period. Additionally, survey responses also show that students experienced positive outcomes as a result of remote learning that included spending increased time on additional learning interests, with family, on self-care, and for dissertation or manuscript writing. Though a variety of supportive resources are already available to students in our institution, results from our survey suggest enhancing these measures and identifying new ones targeted to addressing the academic and emotional needs of PhD students would be beneficial. Such support measures may be appropriate for students in other institutions as well.

## Introduction

The first cases of COVID-19 in the United States (US) were reported toward the end of January 2020, and by mid-March there were widespread school and business closures announced in multiple states [1]. In March 2020, the city of Nashville, TN, home to Vanderbilt University as well as several other leading universities, instituted a broad Stay at Home requirement for all except essential workers [2]. In addition, Vanderbilt University also instituted a stay at home requirement for graduate students, postdocs, faculty, and staff unless they were conducting essential work (a small minority of individuals working on COVID-19 research). There was little time to ramp down research, and most labs closed down in 48 hours, just sufficient time to arrange for care and maintenance of the animals used in research and to terminate ongoing experiments [3]. As this occurred in mid-March, the spring semester was interrupted, and we had to quickly find ways to continue with our educational activities. At the same time, we also had to adapt to new ways of communicating with our students and colleagues.

The Vanderbilt University Interdisciplinary Graduate Program (IGP) and the Quantitative and Chemical Biology (QCB) program are umbrella entry programs for several biomedical science programs/departments at Vanderbilt University, and these programs typically enroll a combined total of 70–80 students each year. First year IGP and QCB students take required coursework to strengthen their core knowledge and complete four 8-week lab rotations. At the end of the first year, students select a thesis research lab and join one of the participating training programs/departments. As students progress through their graduate training, they concentrate on their own individual research projects and continue to satisfy additional requirements set forth by the individual programs/departments, such as advanced coursework, the PhD qualifying exam, and the final dissertation defense.

The COVID-19 pandemic-related university closure caused classes to be moved online and interrupted students’ research and training. Critically, the first year students in our umbrella programs were in their final lab rotation and still involved in selecting their future thesis research labs. Some students had already decided on a lab or were close to a final decision, but about 20% of the class were still uncertain as to which lab they wanted to join. Faculty teaching the interrupted classes quickly adapted to the new demands of remote teaching and individual labs began holding weekly meetings and journal clubs online. Most senior students who had already finished their coursework and were more focused on conducting their thesis research had to adapt to a significant interruption in their research activities.

After the COVID-19-interrupted semester ended, we began to wonder what the effects of these unprecedented circumstances were on our graduate students. In the annual survey we administer at the end of the spring semester, we typically ask the first year graduate students to evaluate their first year in the PhD program. The spring 2020 survey was no different, except we included additional questions asking about these students’ experiences during the university closure. As we began to analyze the results from this survey, we realized that it might prove very valuable to compare the experiences of first year students with those of students who had already completed the first year and were working on a research project (termed “senior students” for the remainder of this paper). Therefore, these senior students were also asked to complete a survey similar to the first year student survey, but with some questions modified to fit the specific circumstances of the senior students. The following is a comparison of the survey results from the first year and senior students, detailing the impact of the COVID-19 pandemic on their educational experience at our institution.

## Methods

Anonymous, voluntary surveys were administered to two populations of students at Vanderbilt University. The first was made up of all 82 first year students in the IGP (65 students) and QCB (10 students) umbrella admission programs, as well as students directly admitted (7 students) to individual departments. The second group was made up of all 441 students who initially entered through one of these three umbrella routes or the MD/PhD program, but who are now in the second year and beyond, and in one of 13 different biomedical departments or programs. All MD/PhD students were in the PhD portion of their curriculum during survey administration.

After survey development, we used Survey Monkey to administer both surveys. The students were asked a variety of multiple choice, rating scale, and open-ended free-text response questions. Students were not required to answer all questions. For quantitative analysis of free-text responses, student answers were grouped into different categories based of the appearance of one or more keywords. The first year student survey was open for students to complete from May 6 to May 22, 2020 (a total of 17 days). The senior student survey was open for students to complete from May 27 to June 12, 2020 (a total of 17 days). Students were sent reminders at the following time points: 1) one week after the survey was administered; 2) four days before the survey was closed; and 3) the day of survey closing. Approval for the study was obtained from the Vanderbilt University Institutional Review Board (IRB 201372 and IRB 201329).

Both surveys asked students questions about their level of stress, the impact of the university closure on remote learning, and their overall psychological health. Surveys also included questions about students’ demographic information including gender, citizenship, and admissions program. Senior students were also asked for their department affiliation, the academic year in which they had started their graduate training, and additional questions about topics more specific to their stage in training, such as the PhD qualifying exam and thesis defense. The surveys used a 6-point Likert scale to allow students to rate their experience with various aspects of university closure and remote learning. The rating scale consisted of six options: 1) extremely high, 2) high, 3) manageable, 4) low, 5) no difference, and 6) not applicable (or “I don’t know”, where appropriate for some questions). For analyses, the scale categories of “extremely high” and “high” were collapsed into “high”.

GraphPad Prism was used to analyze data and generate graphs. Fisher’s exact test was used for all statistical comparisons. Due to the smaller sample size, Fisher’s exact test was used to compare the level of high stress versus all other levels of stress grouped together (manageable, low, no difference and not applicable or I don’t know) for first year and senior students. A similar strategy was used to compare differences in high versus all other levels of negative impact grouped together (manageable, low, no difference and I don’t know).

## Results

### Respondent demographics

First year students in two umbrella admissions programs, first year students directly admitted to individual departments, and senior students in 13 biomedical departments or programs completed the surveys. Respondent demographics for both surveys are shown in Table 1. Survey response rates for the first year and senior student surveys were 96% and 49%, respectively. For both surveys, a majority of respondents were female and were US citizens or legal permanent residents (LPR), which is comparable to the demographics of our overall student population.

**Table 1:** Summary of survey respondents.

	First Year Student Survey	Senior Student Survey
	n (%)	n (%)
**Total survey responses**	79 (96%)	214 (49%)
**Female**	56 (71%)	143 (67%)
**US citizen / LPR**	66 (84%)	189 (88%)

Survey recipients include 82 first year and 441 senior students. Total survey response rate was calculated as the total number of survey responses divided by the number of total survey recipients whereas percent female and US citizen/LPR student response rates were calculated as the total number of female or US citizen/LPR respondents divided by the number of total survey responses.

### Remote learning

Students were asked to list any positive outcomes they had experienced as a result of remote learning during the spring 2020 university closure. Forty-six first year students (58%) and 125 (57%) senior students listed at least one positive outcome resulting from remote learning. Positive outcomes (Table 2) included additional time for learning, spending time with family, focusing on physical and mental health, setting / following a personal schedule, reduced commute, and the ability to attend more seminars virtually. Seven first year and 18 senior students mentioned more than one of these positive outcomes. The differences in positive outcomes reported between first year and senior students were striking in a number of ways, though not particularly surprising given the different stages of their training. A higher proportion of first year students (32.6%) compared to the senior students (19%) reported that the university closure offered them more time to take additional courses, learn new materials and for their research data analysis. Similarly, a higher fraction of first year students (21.7%) felt that they could use the extra time to focus on their physical and mental health. In contrast, a higher proportion of senior students reported that they could attend more seminars, virtually. This is expected as the senior students are likely more aware of various seminars, especially those related to their research focus. An additional positive outcome reported by many senior students was that they had additional time for writing manuscripts and/or their dissertation. Student responses that did not fit in the first ten categories listed in Table 2 and were too varied to group in smaller categories were categorized as “Other”. A small fraction of both the first year and senior respondents reported that they did not experience any positive outcome as a result of remote learning.

**Table 2:** Positive outcomes experienced by students as a result of remote learning.

Positive outcomes of remote learning	First year students,	Senior students,
	n = 46	n = 125
**Additional time for learning**	15 (32.6%)	24 (19%)
**(read papers, data analysis, learn coding / other courses)**		
**Focus on physical and mental health**	10 (21.7%)	11 (8.8%)
**Spend time with family**	6 (13%)	9 (7.2%)
**Set / follow own schedule**	6 (13%)	10 (8%)
**Less commute**	2 (4.3%)	10 (8%)
**Attend more seminars**	2 (4.3%)	16 (12%)
**Additional time for writing manuscript / thesis**	--	22 (17.6%)
**Work-life balance**	--	4 (3.2%)
**More productive**	--	4 (3.2%)
**More communication with thesis advisor**	--	3 (2.4%)
**None**	2 (4.3%)	9 (7.2%)
**Other**	10 (21.7%)	24 (21.6%)

Students’ optional open-ended free-text responses were categorized based on keywords reflective of the positive outcomes they reported. Respondents could mention more than one positive outcome in their response. Seven first year and 18 senior students mentioned more than one positive outcome. The number of responses for each positive outcome is shown, and the percent responses is shown in parentheses.

**Fig 1:**
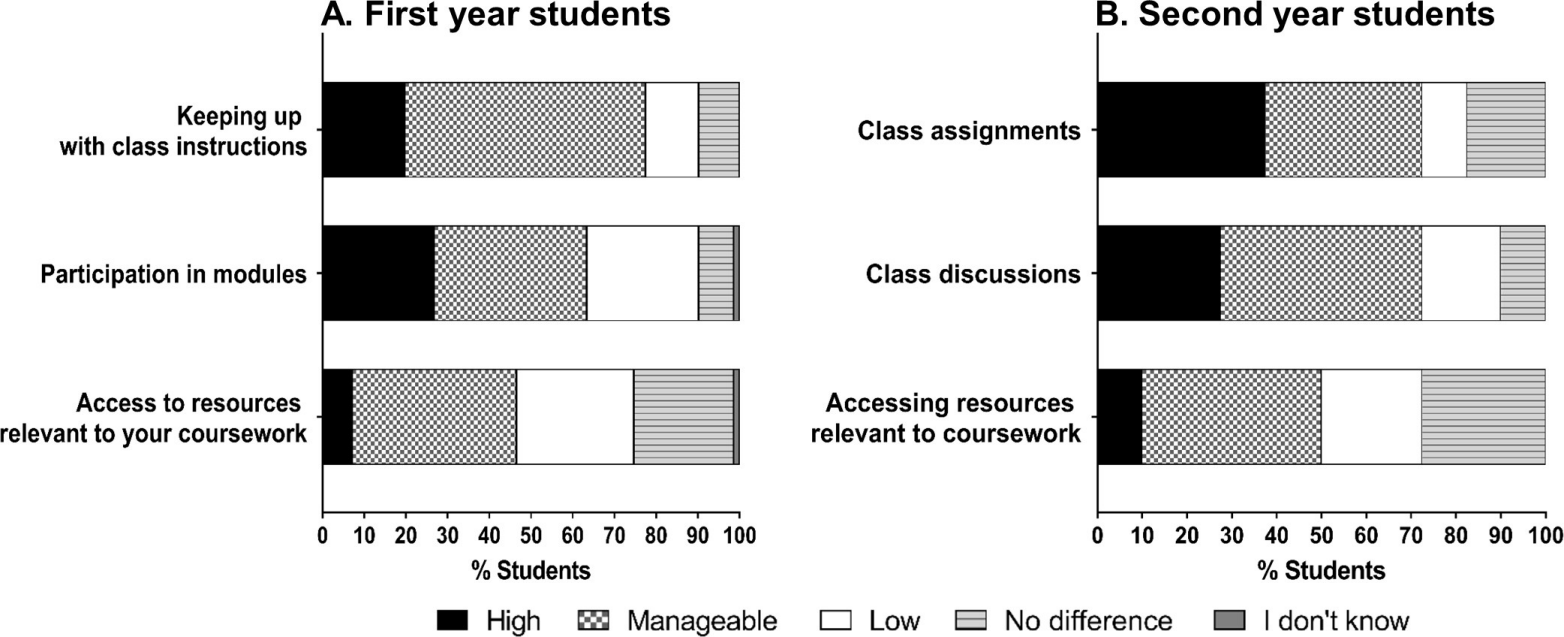
Level of stress with online classes. Students were asked to indicate their level of stress with various aspects of online classes during the remote learning period, compared to in-class learning. Responses shown are from (A) first year (n = 71), and (B) second year students (n = 40) who had classes continuing after the university closed in March 2020. “Modules” in (A) represent the 5-week-long elective courses offered to first year students during the spring semester.

**Fig 2:**
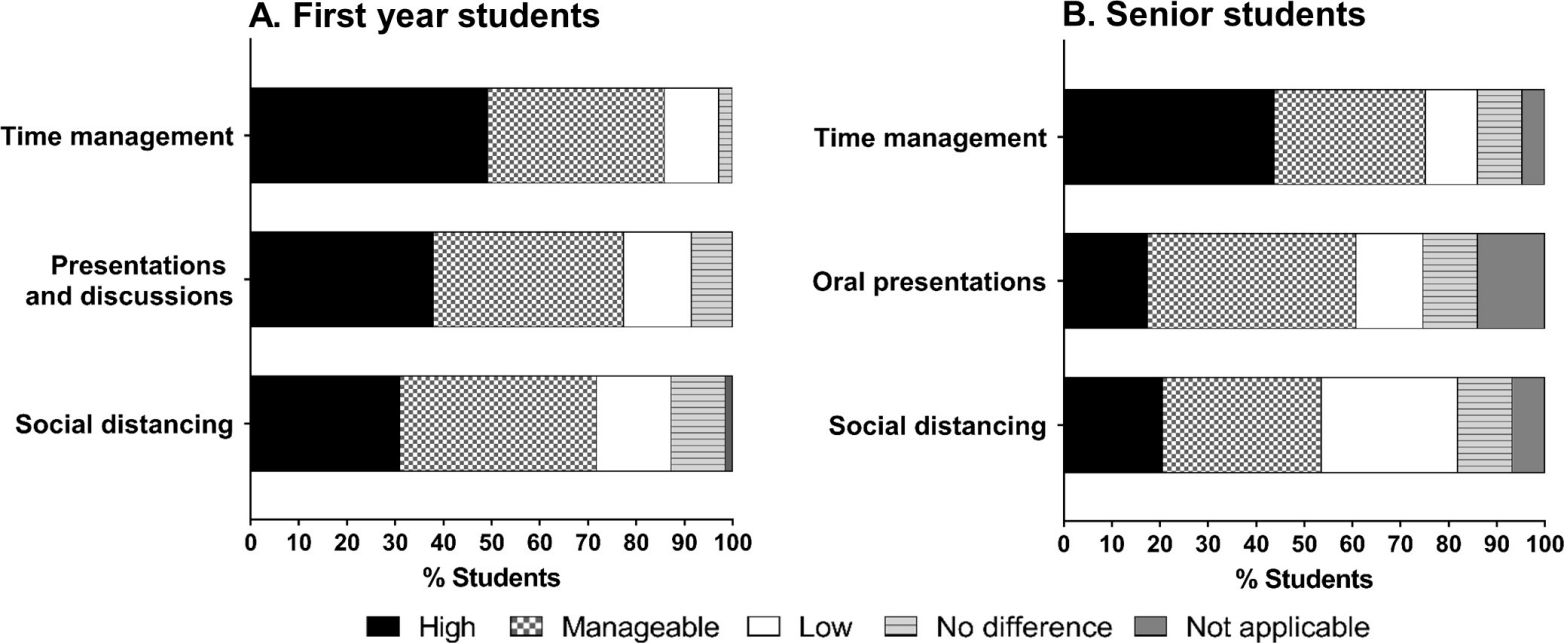
Level of stress associated with other aspects of remote learning. Students were asked to indicate their level of stress with various aspects of remote learning. Responses from (A) first year students (n = 71) and (B) senior students (n = 194) are shown.

A large majority of the first- and the second-year students enrolled in classes during the university closure reported that the level of stress associated with taking online courses was manageable, low, or no different than before the university closure (Fig 1). However, about one-fourth of the first year students reported high stress in participation in modules, which are 5-week-long elective courses. Some second year students also reported high stress in keeping up with class assignments (37.5%) and discussions (27.5%). The aspect of remote learning that was the highest stressor for all students was time management (Fig 2). A substantial fraction of students (49% of first year students, 44% of senior students) reported a high amount of stress associated with time management during the remote learning period. There were no significant gender- or citizenship-based differences in the level of stress with time management in either of the student groups (S1 and S2 Tables). Interestingly, a higher proportion of first year students (38%) as senior students (17.5%) reported high stress with presentations and discussions (p = 0.0009; Fig 2). This is not surprising as the senior students are likely more experienced in presenting scientific materials. Both groups of students have reported comparable level of high stress with social distancing.

Negative aspects of the quarantine were similar between first year and senior students. When students were asked to list the aspects of on-campus learning they missed most during the remote learning period, the most common responses from all students, regardless of seniority level, were interaction with peers and in-person discussions (Table 3). This was unsurprising and in fact, an expected outcome of social distancing. While one in three first year students missed a structured classroom environment, a smaller fraction of senior students (7.6%) missed having a structured research workspace. Likewise, a higher percentage of first year respondents (24.4%) reported that they missed their interaction with instructors and faculty. However, a smaller fraction of senior students (13.2%) also missed interactions with faculty. Presumably the senior students had already established strong relationships with faculty and these were maintained by other types of distance interactions and virtual lab meetings. With quarantine, these early formative interactions were lost or simply not fully developed for first year students, whereas the senior students were likely in a more secure, established position. Perhaps because they are at a more research-intensive stage of graduate training, additional responses from the senior students included that they missed working in the lab/performing experiments, as well as giving in-person scientific presentations and receiving feedback for the same. Student responses that did not fit in the first six categories listed in Table 3 and were too varied to group in smaller categories were categorized as “Other”. Not surprisingly, a higher fraction of the senior students responded that they did not miss any aspect of on-campus learning during the remote learning period.

**Table 3:** Aspects of on-campus learning missed by students.

Aspect of on-campus learning missed	First year students	Senior students
	n = 45	n = 144
Interaction with peers	16 (35.5%)	62 (43%)
In-person discussion	16 (35.5%)	35 (24.3%)
Structured classroom environment / workspace	15 (33.3%)	11 (7.6%)
Interaction with instructors/faculty/advisor	11 (24.4%)	19 (13.2%)
In-person presentations and feedback	1 (2.2%)	11 (7.6%)
Working in the lab/performing experiments	--	24 (16.7%)
None (enjoyed remote learning)	1 (2.2%)	9 (6.2%)
Other	5 (11.1%)	21 (14.6%)

Students’ optional open-ended free-text responses were categorized based on keywords reflective of the aspects of on-campus learning they missed during the remote learning period. Respondents could mention more than one aspect in their response. Fifteen first year and 38 senior students mentioned more than one aspect of on-campus learning that they missed. The number of responses for each positive outcome is shown, and the percent responses is shown in parentheses.

**Fig 3:**
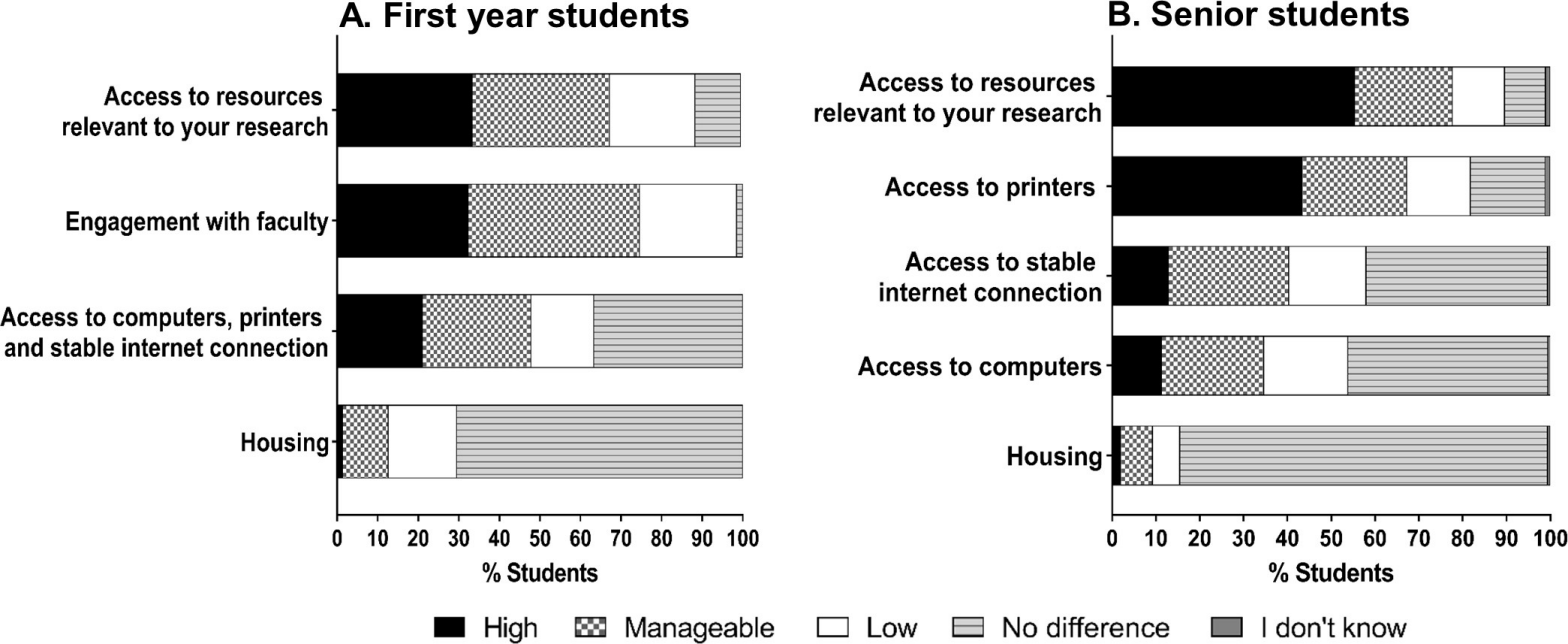
Negative impacts of COVID-19-related university closure. Students were asked to indicate the level of negative impact the university closure had on each item listed. Responses from (A) first year students (n = 71) and (B) senior students (n = 193) are shown.

Lack of access to resources relevant to their research had a high negative impact for all students. The negative impact was higher for senior students (55% high negative impact) compared to first year students (34% high negative impact) (Fig 3). As first year students typically do a series of rotations during the first year, this cohort concentrates on a series of small projects in different labs. In contrast, senior students have chosen a lab and have started their own self-driven, longer-term dissertation research projects. Being kept out of lab prevented students from making meaningful progress on their dissertation research projects during this period, so it is not surprising that senior students rated this as a high negative impact on their graduate training. Fortunately, housing appeared to cause the least amount of stress for all students. International students in our cohort (first year students n = 13; senior students n = 20) did not indicate a high negative impact on housing compared to their peers who are US citizens or LPR (first year students n = 66; senior students n = 194).

### Impact on psychological health

**Fig 4:**
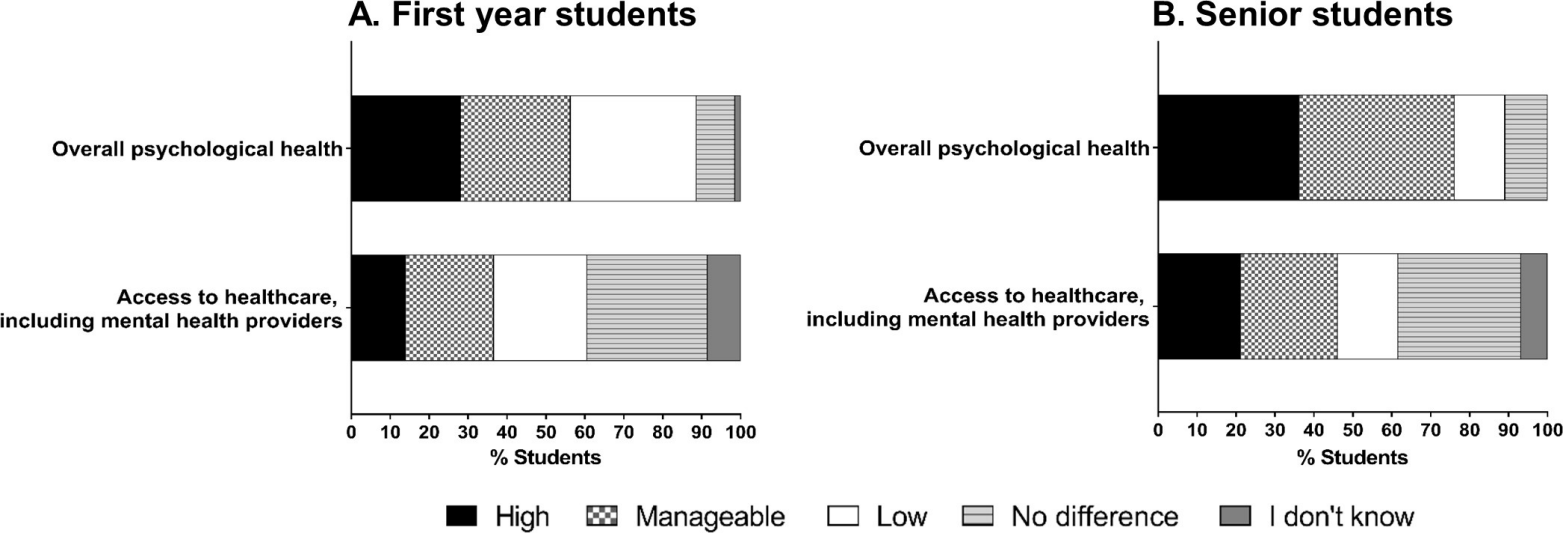
Impact on psychological health. Responses from the (A) first year (n = 71) and (B) senior students (n = 193) when asked to assess the negative impact of the university closure on their overall psychological health, and their access to healthcare, including mental health providers.

Approximately one third of students in all stages of training reported that the university closure had a high negative impact on their overall psychological health (Fig 4). Seventy senior students (36%) reported high negative impact on their overall psychological health. Of these, 66 students were those whose research requires the students to be physically present in the labs to conduct their research (S3 Table). Students who reported a high negative impact on psychological health were more likely to also report a high level of stress associated with classwork, time management, social distancing, and a negative impact on their interactions with peers at Vanderbilt (S1–S3 Figs).

There were no significant differences in overall psychological health associated with gender or citizenship status in the two student groups (S1 and S2 Tables). The availability of physical and mental health services was not impacted for a majority of students from both groups. About 86% of first year and 79% of senior students indicated that their access to healthcare, including to that of mental health providers, was manageable, low or no different during the university closure. A small percentage of senior students (6.7%) were not aware if the university closure impacted their access to healthcare providers.

## Discussion

In response to the unprecedented spread of the COVID-19 outbreak, several universities across the US, including Vanderbilt University, closed their campuses, ramped down ongoing laboratory research activities and transitioned to online teaching. In an attempt to understand the effects of the university closure and resulting circumstances on our graduate students, we surveyed 82 first year, and 441 senior students in the Vanderbilt University Biomedical Sciences PhD programs. We received responses from 96% of first year and almost 49% of senior students with a majority of the responses in both groups coming from female, and US citizen/LPR students, which reflects the demographics of the overall student population in these programs. Survey responses indicate that students experienced both positive and negative outcomes due to the university closure. While positive outcomes of the university closure reported by students included being able to use the additional time away from their labs to enhance their learning in other ways and tending to their physical and/or mental well-being, the most common negative outcomes included high negative impact on their psychological health, stress with time management and lack of interaction with their peers.

### Impact of university closure on perceived psychological health

We were interested in learning what the effects of the pandemic-related university closure were on our students, including any impact to their psychological health. However, we are not trained psychologists, we simply asked students if the university closure combined with the local stay at home order had an effect on their mental health and we did not attempt to parse the nature or the cause of students’ stresses. Indeed, one third of the survey respondents reported a high negative impact on their overall psychological health. This is not surprising, as stay at home orders during the COVID-19 pandemic have resulted in increased stress in the general population, and therefore is not unique to graduate students [4–9]. Nevertheless, a distinct characteristic of a majority of the US and international PhD student population is that they are in a high-pressure, high-stress environment for extended periods of time during their graduate training (typically 5–7 years). Many graduate students find their work stressful, can become frustrated with the work-life balance due to long working hours, incidents of bullying and harassment, and frequently feel uncertain about their future career prospects [10–14]. Prior to the 2020 pandemic, a larger fraction of the graduate student population in US and international institutions was under considerably higher levels of stress, burnout, anxiety and depression compared to the general population [15–19]. Notably, almost half of the graduate students surveyed in a 2014 University of California-Berkeley study reported being depressed [15], and a separate survey of over 2,000 PhD students in various countries showed that there is an overall prevalence of moderate to severe anxiety in 41%, and moderate to severe depression in 39% of the students [18].

In addition to the typical pressures of graduate school, the recent pandemic and stressors related to the ongoing social justice movement are highly concerning as they can take a toll on psychological health of graduate students. We wanted to assess the impact of the pandemic on the typical pressures the graduate students face, especially during critical stages of their graduate training. In this study, there were three groups of students who were at critical junctures in their PhD training at the time of survey administration: 1) first-year students selecting a faculty research mentor; 2) students preparing for, and taking the qualifying exam; and 3) students in the final phase of their graduate training, who were ready to graduate and move to a new position. For first year students, the lack of direct interaction with faculty in the middle of the spring semester was a major loss. Students at this stage of their graduate training typically interact directly and extensively with the faculty both in their classes and more so with faculty in research lab rotations. Approximately 20% of first year students indicated that the interruption resulting from the university closure delayed their decision on their final thesis lab choice. For the students taking the qualifying exam, all 22 students who were scheduled to take one part or the entire exam during the university closure were able to successfully do so virtually. However, four of these students mentioned that the stress caused by the pandemic made it more challenging to prepare for the qualifying exam. In the case of the third group of students, senior students in the final phase of their graduate training, nine respondents successfully defended their final doctoral dissertations, with six doing so remotely via Zoom during the university closure. A positive aspect of these Zoom defenses, as pointed out by the defending students, was that these students were able to invite many friends and extended family members from across the country and around the world to attend the virtual defense.

Most students who defended either shortly before or during the university closure, reported that moving to the next step in their career was interrupted by hiring freezes, travel constraints, and delays to start dates. While students typically experience more pressure as they identify the next step in their careers’ this pressure may make these students especially sensitive to interruptions in wrapping up research projects and uncertainties in the COVID job market. Our survey results, however, show similar levels of overall psychological stress experienced by first year and senior students. A noteworthy observation, though not statistically significant, is that a higher percent of senior students whose research involves predominantly computational work, and who were likely more able to continue their research without interruption while working remotely, reported a lower negative impact to their overall psychological health, compared to those whose research is conducted primarily at the lab bench (S3 Table). Approximately one-third of all of our survey respondents experienced a high negative impact on their psychological health due to the COVID-19 related university closure. Importantly, an event this spring that caused increased stress among many was the killing of George Floyd [20]. The outrage and resulting social justice movement have spread across the country and around the world. The death of Mr. Floyd occurred on May 25, between the close of our first year student survey and the start of our senior student survey, and therefore may have influenced the senior student responses. We did not design the survey to detect any impact of these specific events, but think that it is reasonable to assume that this likely had an impact on the overall level of stress for many students. One aspect of daily life which did not appear to have caused Vanderbilt students significant stress was housing. In a May 2020 survey, nearly half of Berkeley international graduate students expressed concern about their ability to pay for future housing [21]. In contrast, housing was not a source of significant stress for the vast majority of our students.

With no end in sight to the ongoing pandemic, new stay at home orders and / or prolonged periods of remote learning are likely. However, we have thus far been able to return to the research laboratories with essentially no new incidences of campus-transmission due to a broad compliance with approaches designed to minimize infection. In the event of a surge in infection in our research environment, our survey results suggest that we need to consider new ways to provide both academic and emotional support to graduate students to enhance the measures already in place prior to the pandemic. As students might benefit from varied and individualized methods of support, our institution has the following in place to help students reduce their stress and anxiety resulting from the COVID-19 pandemic: appointments via phone, email or zoom with our University Counseling Center, Center for Student Wellbeing and the Graduate Life Coach, a variety of self-directed wellness apps to assist students with meditation, mindfulness, mood tracking, and relaxation techniques [22], along with supplemental group zoom activities for biomedical graduate students such as trivia nights, yoga, and sessions on practicing mindfulness.

### Stress of remote learning

Most students seemed to find the stress associated with online courses during the remote learning period manageable, even though some senior students reported a high level of stress in dealing with assignments and participating in discussions. Overall, the shift to online classes seems to have been accomplished successfully. However, the most surprising result seen in these data is the considerable stress associated with time management. Forty-nine percent of the first year and 44% of the senior student respondents reported a high level of stress associated with time management and this increased to 70–80% for both groups of students that had reported a high negative impact of the university closure on their psychological health. These data are suggestive of a negative correlation between the students’ success with effective time management and the impact on their psychological health. Effective time management has been shown to have a positive impact with better academic outcomes while reducing perceived stress for students [23–26]. It seems quite possible that stress, anxiety or mental health issues could compromise a student’s ability to effectively manage their time though we are unable to determine this conclusively from our survey data. Offering training, workshops or professional development opportunities to enhance time management skills might help students control their time better and alleviate their stress. However, empirical evidence on the effectiveness of time management training on perceived stress remains mixed and scarce [23, 26–28].

It is not entirely clear from our survey data why both groups of students found time management to be stressful. One can speculate that these students were experiencing a very thin line between their academic/professional spaces and their personal spaces during the remote learning, and may have found it difficult to juggle their time between responsibilities at home, tending to their family’s needs, and online classes and related deadlines. The lack of a structured classroom environment or structured workspace might also impact the students’ ability to stay focused and motivated, which could in turn have an impact on their time management and productivity. Findings from our survey show that a higher proportion of first year students (33%) than senior students (8%) also missed having a structured classroom environment or workspace. In a recent survey conducted by the Student Experience in the Research University (SERU) Consortium, 88% of the graduate students experienced at least one obstacle in their transition to remote learning, including a lack of motivation for remote learning (56%), lack of interaction or communication with other students (55%), and a distracting home environment or lack of access to appropriate study space (43%). The SERU COVID-19 survey also found that graduate and professional students were more likely than undergraduates to experience other obstacles in transition to remote learning including serving as the caretaker of one or more of their family members, lack of access to on-campus, research, and library resources, and poor mental health, difficulty concentrating, and increased anxiety and fatigue [29]. Comparably, our survey results show that interaction with their peers and in-person discussions were the top two aspects of on-campus learning that both groups of students missed during the remote learning period.

### Unanticipated benefits of remote learning and quarantine

Our survey results revealed some unanticipated outcomes of the University closure and quarantine, at least in the short term (about 8 weeks). We hypothesized that there might be both positive and negative aspects of the university closure for students whose lives revolved around daily involvement in the laboratory or research group. These survey results supported our hypothesis in that almost 60% of first year and senior students reported some benefits from remote learning and the extended quarantine. Even though students in both groups reported stress with time management, many were able to use time away from the university for self-improvement such as reading papers, attending other online courses or webinars, etc. First year and senior students emphasized spending increased time on health and wellness, time with family, and the opportunity to follow their own schedule as unanticipated benefits. Senior students used the extra time to write up aspects of their work for publication, a stage in training not yet reached by the first year students. It should be stressed that while the quarantine period was relatively short before we began to slowly return to the labs, some of the positive outcomes may well diminish if enforced for a longer time.

## Conclusion

Though the COVID-19 pandemic-related university closure interrupted students’ research progress and impacted their learning and psychological well-being, our study finds that students also experienced some modest positive outcomes as a result of remote learning during the university closure. Students seemed to adapt well to the online classes, but time management was the highest stressor for first year and the senior students alike. While we don’t have a concrete answer from the survey data, first year students most likely realize that deciding on a thesis research lab is critical and they cannot afford a delay in completing this. For the more senior students, time is critical in that a great deal of laboratory experiments need to be performed to complete the PhD requirements and they can ill afford to be sitting at home away from the laboratory. Our study is limited in that we did not design our surveys to identify specific aspects of academic or societal factors that negatively impacted the students’ psychological health, and do not know specifically which aspects contributed to students’ inability to manage their time during the remote learning period. However, what our study finds is that the loss of the research space has a potent negative effect on the social and direct interactions between students and faculty. The success of the biomedical research endeavor is very much dependent on these social interactions, though we perhaps rarely give it the recognition it deserves.

## Supporting information

S1 Table Gender-based impact of university closure on students’ psychological health and time management. (PDF)

S2 Table Citizenship-based impact of university closure on students’ psychological health and time management. (PDF)

S3 Table Negative impact of university closure on overall psychological health for students doing primarily computational research and those doing lab bench research. (PDF)

S4 Table Level of stress with online classes. (PDF)

S5 Table Level of stress associated with other aspects of remote learning. (PDF)

S6 Table Negative impacts of COVID-19-related university closure. (PDF)

S7 Table Impact on psychological health. (PDF)

S1 Fig Level of stress with online classes for students who reported high negative impact of university closure on their psychological health. We analyzed the level of stress with the online classes for students who reported high negative impact of the university closure on their overall psychological health. Within this group, 40% of both the first year (n = 20) and the second year (n = 18) students also reported experiencing high stress in various aspects of their coursework and only 10% of the students experienced high stress in accessing resources relevant to their coursework. “Modules” in “First year students” graph represent the 5-week-long elective courses offered to first year students during the spring semester.
(TIF)

S2 Fig Level of stress with other aspects of remote learning for students who reported high negative impact of university closure on their psychological health. We analyzed the level of stress with various aspects of remote learning, including time management, oral presentations and social distancing, for first year (n = 20) and senior (n = 70) students who reported a high negative impact of the university closure on their overall psychological health. Within this group, time management was the highest stressor for a majority of the students. Additionally, a majority of the first year students within this group also experienced high stress with presentations and discussions as well as social distancing.
(TIF)

S3 Fig Type of impact on interaction with peers and faculty for students who reported high negative impact of university closure on their psychological health. Of the senior students who reported a high negative impact of the university closure on their overall psychological health (n = 70), 74% also reported that they had a negative impact on their interaction with their peers. Fewer students in this group experienced a negative impact on interaction with their thesis advisor (34%) or their department leadership (19%).
(TIF)
