# Correction: Biomedical graduate student experiences during the COVID-19 university closure

**DOI:** 10.1371/journal.pone.0261553

**Published:** 2021-12-14

**Authors:** The PLOS ONE Staff

## Notice of republication

This article was republished on November 24, 2021, to correct errors introduced during typesetting. The conclusion section was erroneously removed. The publisher apologizes for the error. Please download this article again to view the correct version. The originally published, uncorrected article and the republished, corrected articles are provided here for reference.

## Supporting information

S1 File Originally published, uncorrected article. (PDF)

S2 File Republished, corrected article. (PDF)
