# Supplementary material for: Biomedical graduate student experiences during the COVID-19 university closure
Source: PLoS One. 2021 Sep 16;16(9):e0256687. doi: 10.1371/journal.pone.0256687 (PMC8445460; doi:10.1371/journal.pone.0256687)
Supplement: S2 Table — (PDF) [file pone.0256687.s002.pdf]

S2 Table. Citizenship-based impact of university closure on students' psychological health and time management

(A) Students reporting high negative impact to psychological health

	Number of survey responses		Number of students reporting high negative impact to psychological health (percent total)		P value (Fisher's exact test)
	**US citizens/LPR**	**Foreign students**	**US citizens/LPR**	**Foreign students**	
**First year students**	66	13	17 (25.7%)	3 (23.1%)	>0.9999
**Senior students**	189	25	60 (31.7%)	10 (40%)	0.2203

(B) Students reporting high stress with time management

	Number of survey responses		Number of students reporting high stress with time management (percent total)		P value (Fisher's exact test)
	**US citizens/LPR**	**Foreign students**	**US citizens/LPR**	**Foreign students**	
**First year students**	66	13	33 (50%)	2 (15.4%)	0.0847
**Senior students**	189	25	75 (39.7%)	10 (40%)	0.6368
