# Supplementary material for: Biomedical graduate student experiences during the COVID-19 university closure
Source: PLoS One. 2021 Sep 16;16(9):e0256687. doi: 10.1371/journal.pone.0256687 (PMC8445460; doi:10.1371/journal.pone.0256687)
Supplement: S3 Table — (PDF) [file pone.0256687.s003.pdf]

S3 Table. Negative impact of University closure on overall psychological health for senior students doing primarily computational research and those doing lab bench research

Senior Students' research type	Number of senior student survey responses	Number (percent) of senior students reporting high negative impact on psychological health
Computational research	23	4 (17.4%)
Lab bench research	196	66 (33.7%)

P value = 0.1559
