# Supplementary material for: Biomedical graduate student experiences during the COVID-19 university closure
Source: PLoS One. 2021 Sep 16;16(9):e0256687. doi: 10.1371/journal.pone.0256687 (PMC8445460; doi:10.1371/journal.pone.0256687)
Supplement: S4 Table — (PDF) [file pone.0256687.s004.pdf]

S4 Table. Level of stress with online classes. Values used to create Fig 1 are shown below. Students were asked to indicate their level of stress with various aspects of online classes during the remote learning period, compared to in-class learning. Responses shown are from (A) first year (n=71), and (B) second year students (n=40) who had classes continuing after the university closed in March 2020. "Modules" in (A) represent the 5-week-long elective courses offered to first year students during the spring semester.

(A) First year students (n=71)	High n(%)	Manageable n(%)	Low n(%)	No difference n(%)	I don't know n(%)
Keeping up with class instructions	14(19.7%)	41(57.8%)	9(12.7%)	7(9.9%)	0(0%)
Participation in modules	19(26.8%)	26(36.6%)	19(26.8%)	6(8.5%)	1(1.4%)
Access to resources relevant to your coursework	5(7%)	28(39.4%)	20(28.2%)	17(23.9%)	1(1.4%)

(B) Second year students (n=40)	High n(%)	Manageable n(%)	Low n(%)	No difference n(%)	I don't know n(%)
Class assignments	15(37.5%)	14(35%)	4(10%)	7(17.5%)	0(0%)
Class discussions	11(27.5%)	18(45%)	7(17.5%)	4(10%)	0(0%)
Accessing resources relevant to coursework	4(10%)	16(40%)	9(22.5%)	11(27.5%)	0(0%)
