# Supplementary material for: Biomedical graduate student experiences during the COVID-19 university closure
Source: PLoS One. 2021 Sep 16;16(9):e0256687. doi: 10.1371/journal.pone.0256687 (PMC8445460; doi:10.1371/journal.pone.0256687)
Supplement: S5 Table — (PDF) [file pone.0256687.s005.pdf]

S5 Table. Level of stress associated with other aspects of remote learning. Values used to create Fig 2 are shown below. Students were asked to indicate their level of stress with various aspects of remote learning. Responses from (A) first year students (n=71) and (B) senior students (n=194) are shown.

**(A) First year students (n=71)**	**High n(%)**	**Manageable n(%)**	**Low n(%)**	**No difference n(%)**	**Not applicable n(%)**
Time management	35(49.3%)	26(36.6%)	8(11.3%)	2(2.8%)	0(0%)
Presentations and discussions	27(38%)	28(39.4%)	10(14.1%)	6(8.5%)	0(0%)
Social distancing	22(31%)	29(40.9%)	11(15.5%)	8(11.3%)	1(1.4%)

**(B) Senior students (n=194)**	**High n(%)**	**Manageable n(%)**	**Low n(%)**	**No difference n(%)**	**Not applicable n(%)**
Time management	85(43.8%)	61(31.4%)	21(10.8%)	18(9.3%)	9(4.6%)
Oral presentations	34(17.5%)	84(43.3%)	27(13.9%)	22(11.3%)	27(13.9%)
Social distancing	40(20.6%)	64(33%)	55(28.4%)	22(11.3%)	13(6.7%)
