# Supplementary material for: Biomedical graduate student experiences during the COVID-19 university closure
Source: PLoS One. 2021 Sep 16;16(9):e0256687. doi: 10.1371/journal.pone.0256687 (PMC8445460; doi:10.1371/journal.pone.0256687)
Supplement: S6 Table — (PDF) [file pone.0256687.s006.pdf]

S6 Table. Negative impacts of COVID-19-related university closure. Values used to create Fig 3 are shown below. Students were asked to indicate the level of negative impact the university closure had on each item listed. Responses from (A) first year students (n=71) and (B) senior students (n=193) are shown.

(A) First year students (n=71)	High n(%)	Manageable n(%)	Low n(%)	No difference n(%)	I don't know n(%)
Access to resources relevant to your research	24(33.8%)	24(33.8%)	15(21.1%)	8(11.3%)	0(0%)
Engagement with faculty	23(32.4%)	30(42.3%)	17(23.9%)	1(1.4%)	0(0%)
Access to computers, printers and stable internet connection	15(21.1%)	19(26.8%)	11(15.5%)	26(36.6%)	0(0%)
Housing	1(1.4%)	8(11.3%)	12(16.9%)	50(70.4%)	0(0%)

(B) Senior students (n=193)	High n(%)	Manageable n(%)	Low n(%)	No difference n(%)	I don't know n(%)
Access to resources relevant to your research	107(55.4%)	43(22.3%)	23(11.9%)	18(9.3%)	2(1%)
Access to printers	84(43.5%)	46(23.8%)	28(14.5%)	33(17.1%)	2(1%)
Access to stable internet connection	25(13%)	53(27.5%)	34(17.6%)	80(41.5%)	1(0.5%)
Access to computers	22(11.4%)	45(23.3%)	37(19.2%)	88(45.6%)	1(0.5%)
Housing	4(2.1%)	14(7.3%)	12(6.2%)	162(83.9%)	1(0.5%)
