# Supplementary material for: Biomedical graduate student experiences during the COVID-19 university closure
Source: PLoS One. 2021 Sep 16;16(9):e0256687. doi: 10.1371/journal.pone.0256687 (PMC8445460; doi:10.1371/journal.pone.0256687)
Supplement: S7 Table — (PDF) [file pone.0256687.s007.pdf]

S7 Table. Impact on psychological health. Values used to create Fig 4 are shown below. Responses from the (A) first year (n=71) and (B) senior students (n=193) when asked to assess the negative impact of the university closure on their overall psychological health, and their access to healthcare, including mental health providers.

**(A) First year students (n=71)**	**High n(%)**	**Manageable n(%)**	**Low n(%)**	**No difference n(%)**	**I don't know n(%)**
Overall psychological health	20(28.2%)	20(28.2%)	23(32.4%)	7(9.9%)	1(1.4%)
Access to healthcare, including mental health providers	10(14.1%)	16(22.5%)	17(23.9%)	22(31%)	6(8.5%)

**(B) Senior students (n=193)**	**High n(%)**	**Manageable n(%)**	**Low n(%)**	**No difference n(%)**	**I don't know n(%)**
Overall psychological health	70(36.3%)	77(39.9%)	25(13%)	21(10.9%)	0(0%)
Access to healthcare, including mental health providers	41(21.2%)	48(24.9%)	30(15.5%)	61(31.6%)	13(6.7%)
