# Supplementary figures and images for: Biomedical graduate student experiences during the COVID-19 university closure
Source: PLoS One. 2021 Sep 16;16(9):e0256687. doi: 10.1371/journal.pone.0256687 (PMC8445460; doi:10.1371/journal.pone.0256687)

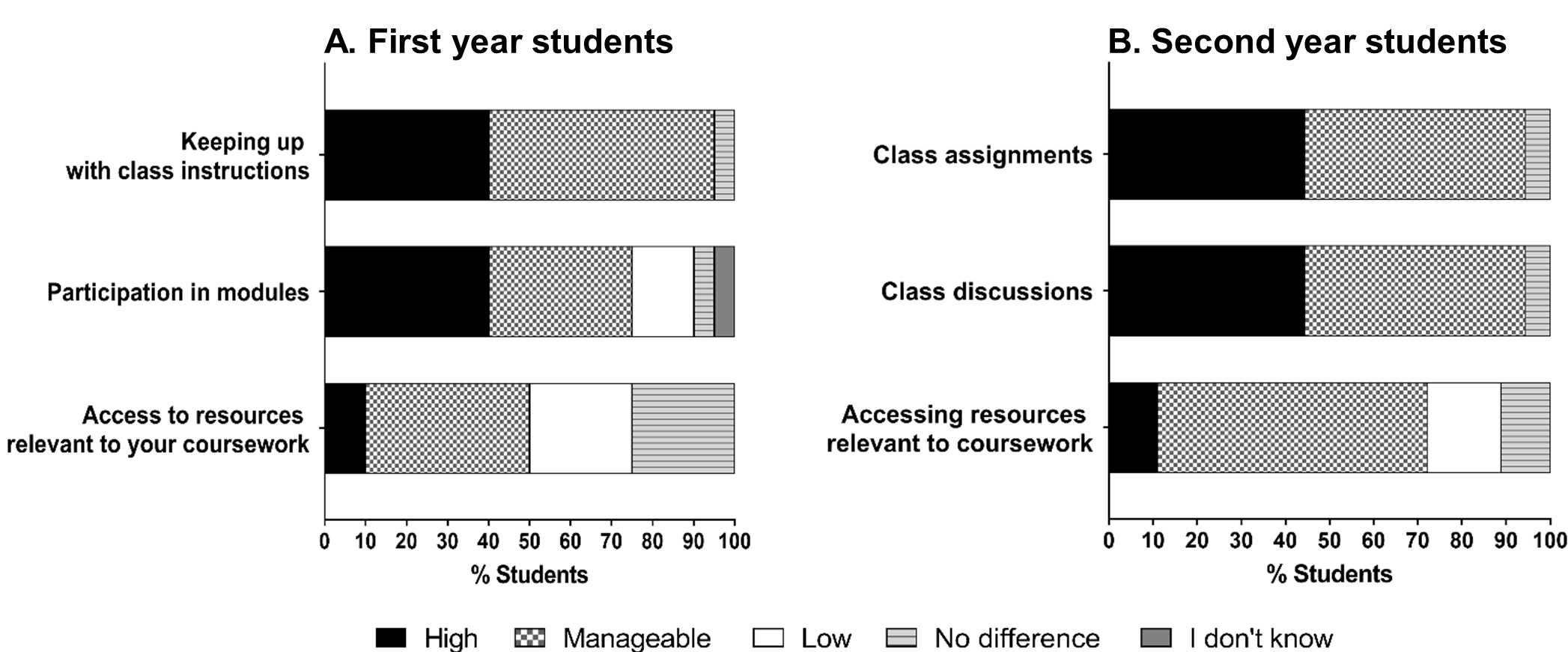

Supplement: S1 Fig — We analyzed the level of stress with the online classes for students who reported high negative impact of the university closure on their overall psychological health. Within this group, 40% of both the first year (n = 20) and the second year (n = 18) students also reported experiencing high stress in various aspects of their coursework and only 10% of the students experienced high stress in accessing resources relevant to their coursework. “Modules” in “First year students” graph represent the 5-week-long elective courses offered to first year students during the spring semester. (TIF) [file pone.0256687.s008.tif]

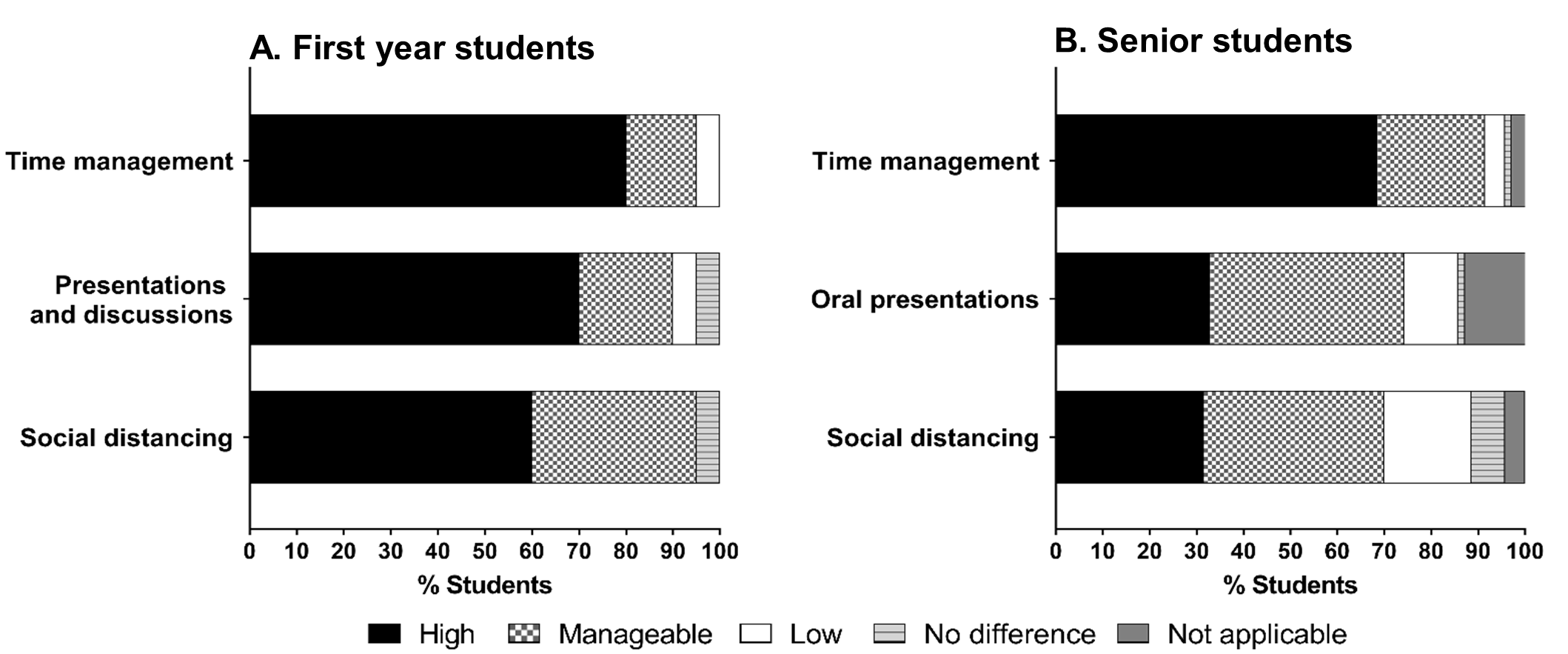

Supplement: S2 Fig — We analyzed the level of stress with various aspects of remote learning, including time management, oral presentations and social distancing, for first year (n = 20) and senior (n = 70) students who reported a high negative impact of the university closure on their overall psychological health. Within this group, time management was the highest stressor for a majority of the students. Additionally, a majority of the first year students within this group also experienced high stress with presentations and discussions as well as social distancing. (TIF) [file pone.0256687.s009.tif]

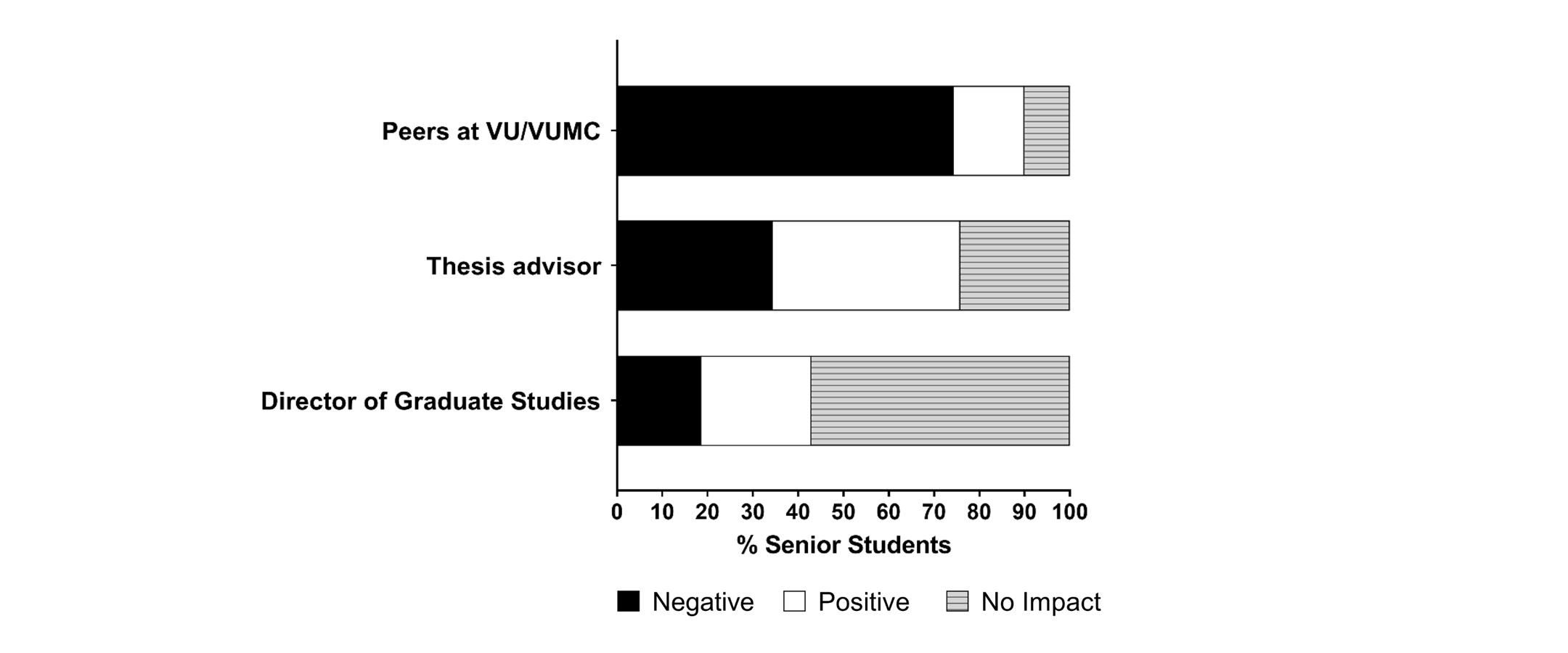

Supplement: S3 Fig — Of the senior students who reported a high negative impact of the university closure on their overall psychological health (n = 70), 74% also reported that they had a negative impact on their interaction with their peers. Fewer students in this group experienced a negative impact on interaction with their thesis advisor (34%) or their department leadership (19%). (TIF) [file pone.0256687.s010.tif]
